# Supplementary material for: Development and Validation of an Insulin Resistance Model for a Population with Chronic Kidney Disease Using a Machine Learning Approach
Source: Nutrients. 2022 Jul 9;14(14):2832. doi: 10.3390/nu14142832 (PMC9319821; doi:10.3390/nu14142832)
Supplement: Supplementary file 1 [file nutrients-14-02832-s001.zip › nutrients-1792320-supplementary.pdf]

Supplementary data

Table S1. All baseline characteristics according to train, test and validation group.

	Overall	Train	Validation	Test	p-value
**Case number, n**	**1229**	**675**	**185**	**369**	
**HOMA_IR**	4.73(4.21-5.25)	4.74(4.07-5.41)	5.15(3.73-6.56)	4.52(3.67-5.37)	<.0001
**Age (y/o)**	71.51(70.7-72.33)	71.54(70.49-72.59)	71.97(70.38-73.55)	71.25(69.69-72.81)	<.0001
**Male, n (%)**	573(40.99)	320(41.83)	95(44.4)	158(37.85)	0.3543
**Ethnicity, n (%)**					
**Non-Hispanic white**	824(82.62)	438(80.78)	128(84.09)	258(85.17)	0.2082
**Non-Hispanic black**	214(9.24)	125(9.75)	30(9.36)	59(8.29)	
**Mexican American/others**	191(8.13)	112(9.47)	27(6.55)	52(6.54)	
**Body Mass Index (kg/m²)**	29.04(28.61-29.47)	29(28.35-29.65)	29.72(28.43-31)	28.79(28.01-29.57)	<.0001
**Laboratory data**					
**Glycohemoglobin (%)**	5.96(5.88-6.03)	5.92(5.84-6.01)	6.08(5.87-6.3)	5.95(5.84-6.07)	<.0001
**Fasting plasma glucose (mg/dl)**	109.75(107.05-112.46)	108.99(105.87-112.1)	113.82(103.49-124.16)	109.12(105.09-113.16)	<.0001
**HDL-cholesterol (mg/dL)**	53.06(52.06-54.06)	53.25(51.81-54.7)	53.83(51.52-56.14)	52.35(50.2-54.51)	<.0001
**Total cholesterol (mg/dL)**	193.47(190.44-196.51)	193.71(190.3-197.12)	196.91(187.7-206.11)	191.38(185.16-197.6)	<.0001
**Triglycerides (mg/dL)**	149.91(143.58-156.24)	149.13(139.2-159.07)	151.77(136.86-166.67)	150.38(138.77-161.98)	<.0001
**Daily food intake**					
**Daily protein intake (gm)/kg**	0.85(0.82-0.89)	0.85(0.81-0.89)	0.86(0.79-0.92)	0.86(0.8-0.91)	<.0001
**Daily energy intake (kcal)/kg**	21.82(21.19-22.46)	22.02(21.1-22.94)	21.39(19.94-22.84)	21.68(20.38-22.97)	<.0001
**Daily protein intake ratio (%)**	16.06(15.62-16.49)	15.82(15.24-16.41)	16.7(15.88-17.52)	16.15(15.49-16.82)	<.0001
**Daily carbohydrate intake ratio (%)**	50.15(49.29-51.01)	50.24(49-51.49)	49.19(47.86-50.52)	50.45(49.16-51.73)	<.0001
**Daily total fat intake ratio (%)**	33.8(33.18-34.41)	33.93(33.01-34.85)	34.11(32.76-35.47)	33.4(32.37-34.43)	<.0001
**Daily fiber intake (gm)**	13.82(13.22-14.42)	13.56(12.84-14.28)	14.6(12.96-16.24)	13.9(12.7-15.09)	<.0001
**Daily cholesterol intake (mg)**	241.11(227.22-255)	253.91(230.81-277.01)	230.45(208.21-252.68)	223.66(200.49-246.82)	<.0001
**Total Folate (mcg)**	340.79(328.33-353.26)	335.77(318.32-353.22)	375.69(338.37-413.01)	332.65(312.63-352.68)	<.0001
**Total saturated fatty acids (gm)**	20.98(20.15-21.81)	21.22(20.01-22.44)	21.39(19.42-23.35)	20.34(18.77-21.92)	<.0001
**Total monounsaturated fatty acids (gm)**	23.2(22.27-24.12)	23.39(21.99-24.78)	22.94(21.1-24.78)	22.98(21.3-24.66)	<.0001
**Total polyunsaturated fatty acids (gm)**	13.92(13.36-14.49)	14.08(13.21-14.95)	13.22(12.03-14.41)	14(12.82-15.17)	<.0001
**Vitamin B6 (mg)**	1.69(1.62-1.76)	1.66(1.56-1.75)	1.83(1.63-2.02)	1.68(1.55-1.8)	<.0001
**Vitamin B12 (mcg)**	4.66(4.34-4.98)	4.54(4.15-4.92)	5.33(4.61-6.04)	4.56(3.78-5.34)	<.0001
**Vitamin C (mg)**	78.24(72.81-83.67)	77.44(69.78-85.09)	76.46(63.04-89.87)	80.52(72.29-88.76)	<.0001
**Calcium (mg)**	760.8(731.17-790.44)	762.7(723.99-801.4)	810.63(722.34-898.93)	733.14(679.76-786.52)	<.0001
**Phosphorus (mg)**	1083.49(1050.23-1116.74)	1086.12(1042.32-1129.92)	1108.97(1037.8-1180.14)	1066.38(1002.84-1129.93)	<.0001
**Magnesium (mg)**	238.5(230.36-246.64)	237.12(227.04-247.21)	246.6(231.36-261.84)	236.99(221.06-252.92)	<.0001

**Iron (mg)**	13.57(13.03-14.1)	13.49(12.79-14.19)	14.97(13.47-16.47)	13.02(12.14-13.89)	<.0001
**Zinc (mg)**	9.99(9.49-10.49)	9.74(9.17-10.32)	11.95(10.14-13.76)	9.48(8.8-10.15)	<.0001
**Copper (mg)**	1.08(1.03-1.13)	1.05(1-1.09)	1.11(0.99-1.23)	1.12(1.01-1.24)	<.0001
**Sodium (mg)**	2782.85(2687.8-2877.9)	2811.09(2675.21-2946.97)	2756.21(2569.42-2943)	2745.85(2573.55-2918.15)	<.0001
**Potassium (mg)**	2366.12(2298.49-2433.75)	2331.67(2242.45-2420.89)	2474.48(2315.69-2633.26)	2374.24(2246.74-2501.74)	<.0001
**Selenium (mcg)**	88.69(85.61-91.78)	89.13(84.83-93.44)	90.79(84.66-96.92)	86.9(81.36-92.44)	<.0001
**Caffeine (mg)**	144.84(131.87-157.81)	148.43(134.74-162.12)	165.36(129.11-201.61)	128.48(105.53-151.42)	<.0001
**Theobromine (mg)**	30.68(26.29-35.07)	30.8(24.45-37.15)	24.2(15.31-33.08)	33.62(26.2-41.04)	<.0001
**Alcohol (gm)**	4.05(3.12-4.99)	4.62(3.26-5.99)	4.72(2.05-7.39)	2.73(1.54-3.91)	<.0001
**Smoking, n (%)**	606(49.67)	338(50.46)	86(46.59)	182(49.77)	0.6941
**Cardiovascular disease, n (%)**	188(16.48)	109(18.79)	35(19.34)	44(11.01)	0.0073
**Hypertension, n (%)**	882(70.82)	484(70.76)	133(75.09)	265(68.86)	0.5049
**Diabetes mellitus, n (%)**	425(30.81)	223(29.27)	67(35.81)	135(31.08)	0.3706
**Case number, n**	572(44.51)	314(44.94)	86(44.2)	172(43.9)	0.9625

Table S2. Unweighted data of descriptive statistics in all baseline characteristics according to insulin resistance (IR>3) or not (IR ≤3).

	**Overall**	**HOMA IR ≤ 3**	**HOMA IR >3**	**p-value**
**Epidemiology**				
Case number, n	**1229**	657	572	
HOMA-IR	4.96±8.33	1.73±0.69	8.68±11.08	<.0001
Age (y/o)	73.39±10.24	74.61±10.57	71.99±9.66	<.0001
Male, n (%)	573(46.62)	290(44.14)	283(49.48)	0.0614
Ethnicity, n (%)				
Non-Hispanic white	824(67.05)	475(72.3)	349(61.01)	0.0001
Non-Hispanic black	214(17.41)	95(14.46)	119(20.8)	
Mexican American/others	191(15.54)	87(13.24)	104(18.18)	
Body Mass Index (kg/m^2)	28.97±6.39	26.65±5.04	31.63±6.73	<.0001
**Laboratory data**				
Glycohemoglobin (%)	6.03±1.08	5.72±0.69	6.4±1.3	<.0001
Fasting plasma glucose (mg/dl)	112.16±38.5	98.27±18.69	128.11±48.07	<.0001
HDL-cholesterol (mg/dL)	52.83±16.5	56.83±17.11	48.25±14.5	<.0001
Total cholesterol (mg/dL)	192.24±47.81	195.96±49.92	187.96±44.92	0.0032
Triglycerides (mg/dL)	147.68±96.86	123.51±61.53	175.43±119.93	<.0001
**Daily food intake**				
**Macronutrients**				
Daily energy intake (kcal)/kg	21.46±9.72	23.24±10.35	19.41±8.5	<.0001
Daily carbohydrate intake ratio (%)	50.36±10.07	51.7±9.6	48.82±10.38	<.0001
Daily protein intake (gm)/kg	0.84±0.43	0.88±0.44	0.79±0.41	0.0002
Daily protein intake ratio (%)	16.07±5.16	15.62±5.08	16.58±5.21	0.0012
Daily total fat intake ratio (%)	33.57±8.76	32.68±8.31	34.6±9.16	0.0001
Total saturated fatty acids (gm)	20.11±12.15	19.52±12.06	20.79±12.22	0.0677
Total monounsaturated fatty acids (gm)	22.59±13.7	21.64±13.32	23.69±14.06	0.0089
Total polyunsaturated fatty acids (gm)	13.56±8.92	13.19±8.74	13.98±9.11	0.1214
Daily cholesterol intake (mg)	242.82±194.27	223.16±188.75	265.39±198.2	0.0001
**Micronutrients**				
Daily fiber intake (gm)	13.74±7.81	13.93±7.9	13.52±7.69	0.3615
Total Folate (mcg)	331.9±187.91	336.98±186.77	326.07±189.22	0.3101
Vitamin B6 (mg)	1.63±1.05	1.65±1.03	1.61±1.06	0.5898
Vitamin B12 (mcg)	4.39±4.41	4.33±4.11	4.46±4.73	0.6021
Vitamin C (mg)	78.44±80.03	79.1±73.24	77.68±87.24	0.7589
Calcium (mg)	733.64±458.75	727.33±449.13	740.88±469.86	0.6056

Phosphorus (mg)	1054.18±481.47	1032.47±472.04	1079.1±491.3	0.0903
Magnesium (mg)	233.4±112.58	233.71±111.51	233.05±113.9	0.9185
Iron (mg)	13.25±7.58	13.43±7.77	13.04±7.36	0.3733
Zinc (mg)	9.66±7.55	9.48±8.32	9.87±6.54	0.3564
Copper (mg)	1.06±0.71	1.05±0.64	1.08±0.79	0.4757
Sodium (mg)	2696.5±1340.68	2620.94±1283.36	2783.29±1399.72	0.0353
Potassium (mg)	2291.13±1045.91	2282.81±1011.44	2300.69±1084.95	0.7652
Selenium (mcg)	87.36±50.49	85.38±53.13	89.64±47.23	0.1369
Caffeine (mg)	126.8±150.51	125.43±143.56	128.36±158.22	0.7352
Theobromine (mg)	29.39±62.27	30.47±65.05	28.16±58.96	0.514
Alcohol (gm)	3.66±13.34	4.35±13.86	2.87±12.67	0.0515
Smoking, n (%)	606(49.31)	322(49.01)	284(49.65)	0.823
Cardiovascular disease, n (%)	188(15.3)	92(14)	96(16.78)	0.1768
Hypertension, n (%)	882(72)	423(64.68)	459(80.39)	<.0001
Diabetes mellitus, n (%)	425(34.58)	126(19.18)	299(52.27)	<.0001

Figure S1. SHAP values of all features from XGBoost algorithm

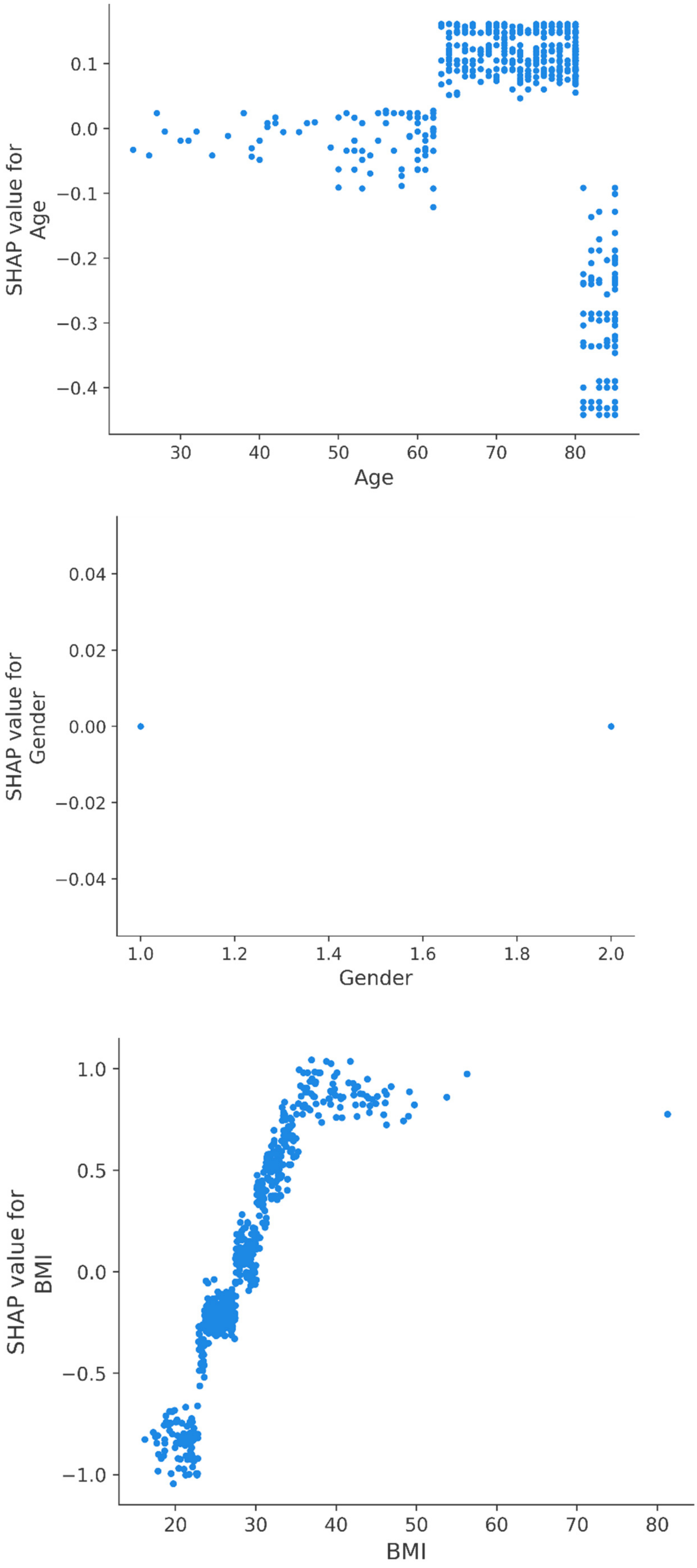

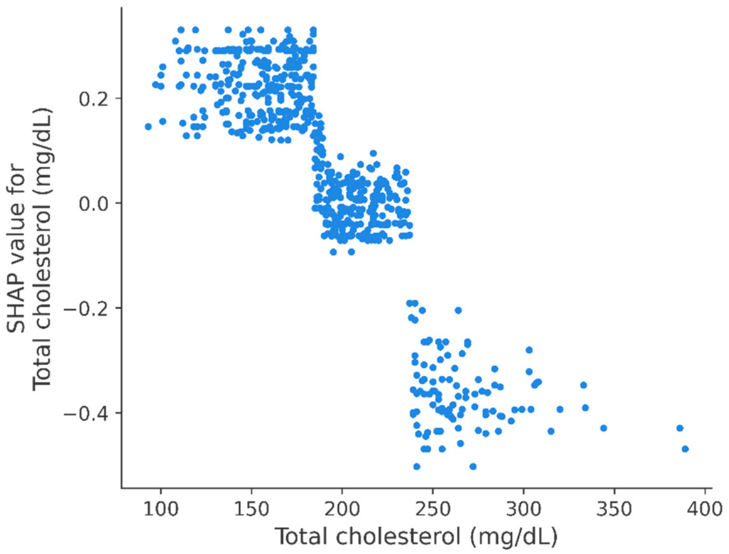
SHAP value for
Total cholesterol (mg/dL)
0.2
0.0
−0.2
−0.4
100
150
200
250
300
350
400
Total cholesterol (mg/dL)

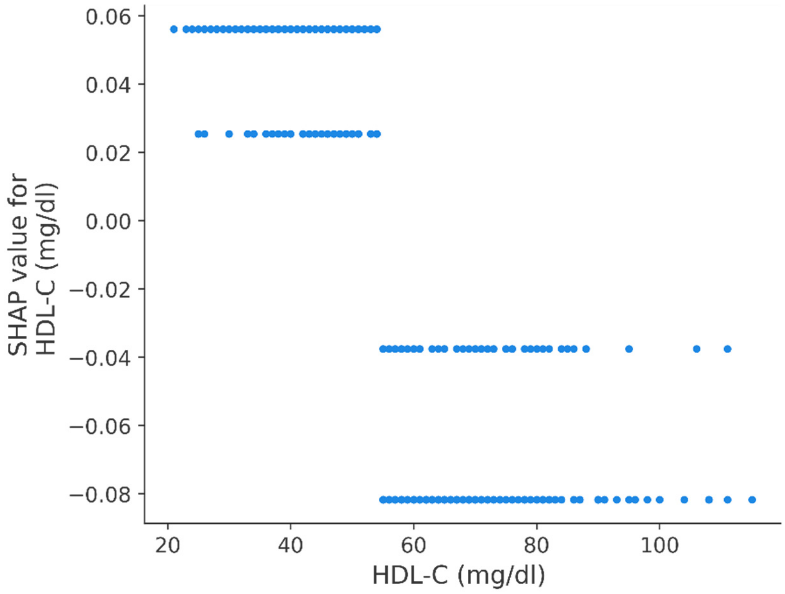
SHAP value for
HDL-C (mg/dl)
0.06
0.04
0.02
0.00
−0.02
−0.04
−0.06
−0.08
20
40
60
80
100
HDL-C (mg/dl)

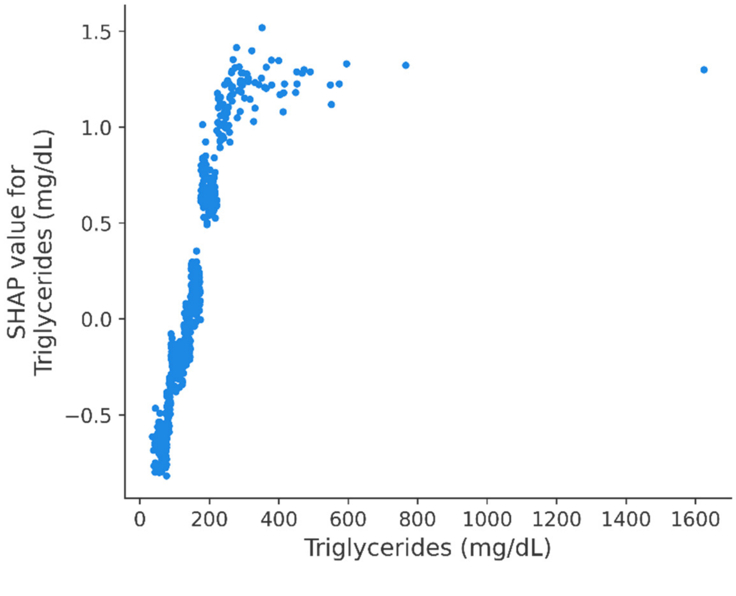
SHAP value for
Triglycerides (mg/dL)
1.5
1.0
0.5
0.0
−0.5
0
200
400
600
800
1000
1200
1400
1600
Triglycerides (mg/dL)

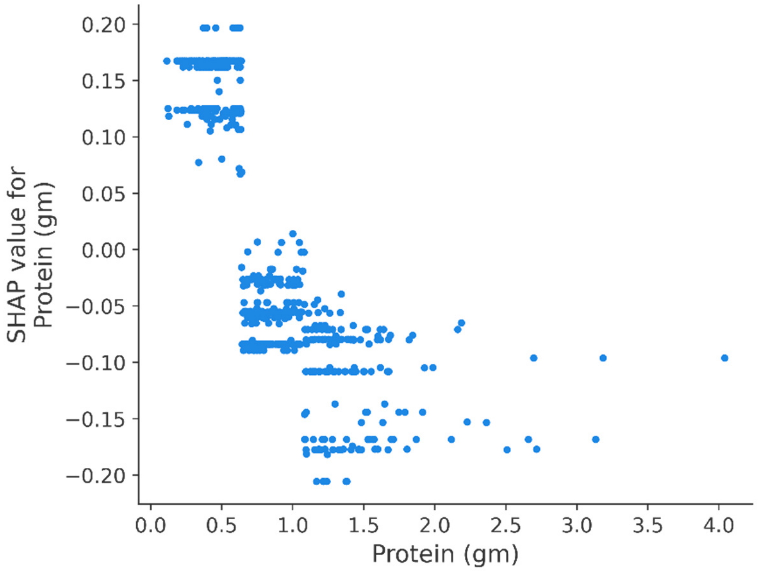
0.20
0.15
0.10
0.05
0.00
−0.05
−0.10
−0.15
−0.20
SHAP value for
Protein (gm)
0.0
0.5
1.0
1.5
2.0
2.5
3.0
3.5
4.0
Protein (gm)

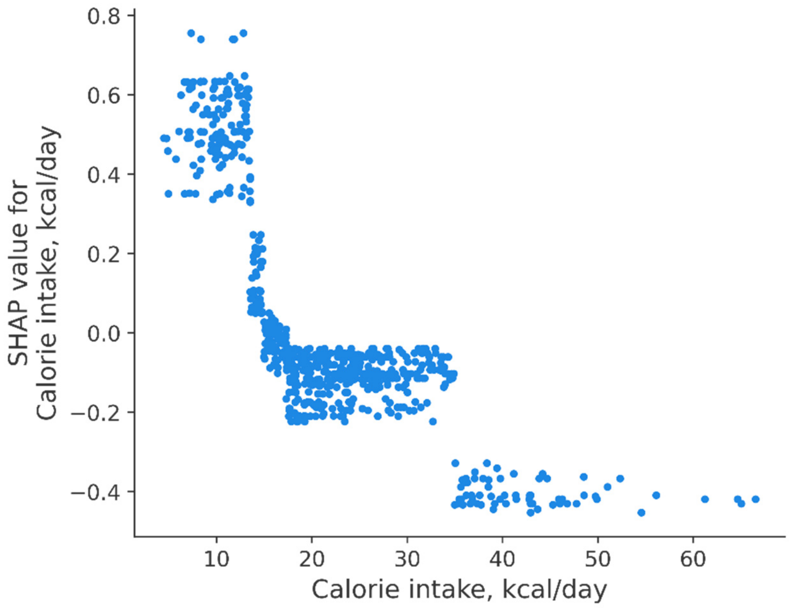
0.8
0.6
0.4
0.2
0.0
−0.2
−0.4
SHAP value for
Calorie intake, kcal/day
10
20
30
40
50
60
Calorie intake, kcal/day

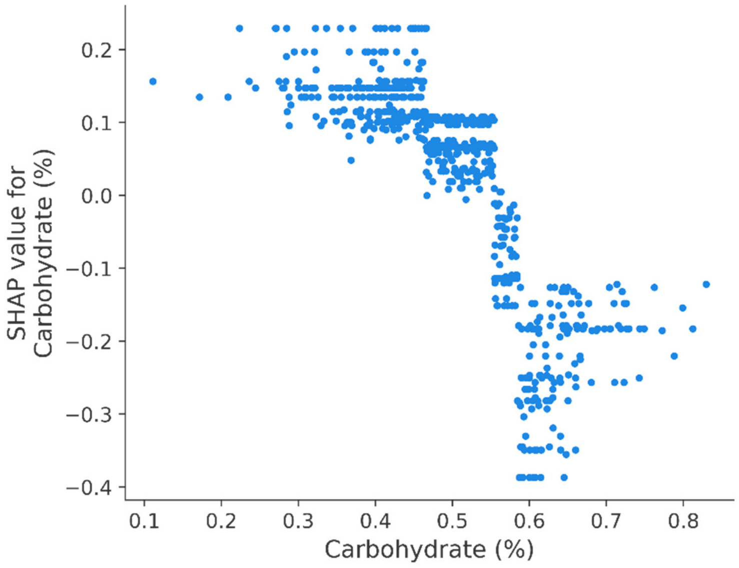
0.2
0.1
0.0
−0.1
−0.2
−0.3
−0.4
SHAP value for
Carbohydrate (%)
0.1
0.2
0.3
0.4
0.5
0.6
0.7
0.8
Carbohydrate (%)

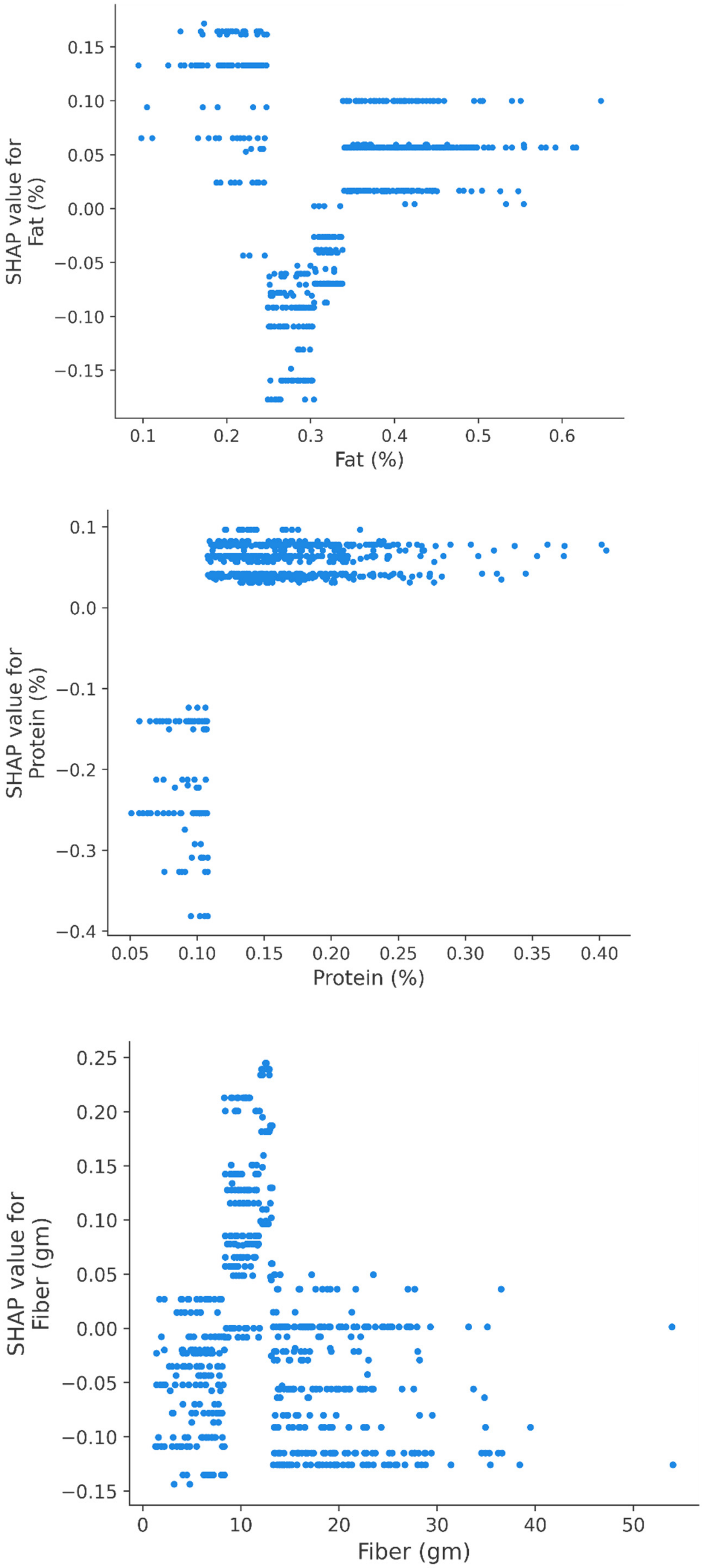
SHAP value for
Fat (%)
0.15
0.10
0.05
0.00
−0.05
−0.10
−0.15
0.1
0.2
0.3
0.4
0.5
0.6
Fat (%)
SHAP value for
Protein (%)
0.1
0.0
−0.1
−0.2
−0.3
−0.4
0.05
0.10
0.15
0.20
0.25
0.30
0.35
0.40
Protein (%)
SHAP value for
Fiber (gm)
0.25
0.20
0.15
0.10
0.05
0.00
−0.05
−0.10
−0.15
0
10
20
30
40
50
Fiber (gm)

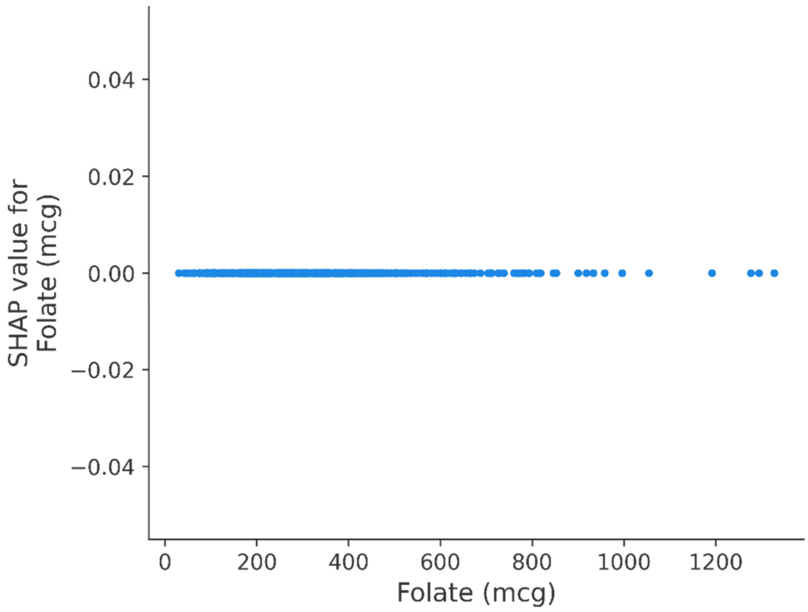
SHAP value for
Folate (mcg)
0.04
0.02
0.00
−0.02
−0.04
0
200
400
600
800
1000
1200
Folate (mcg)

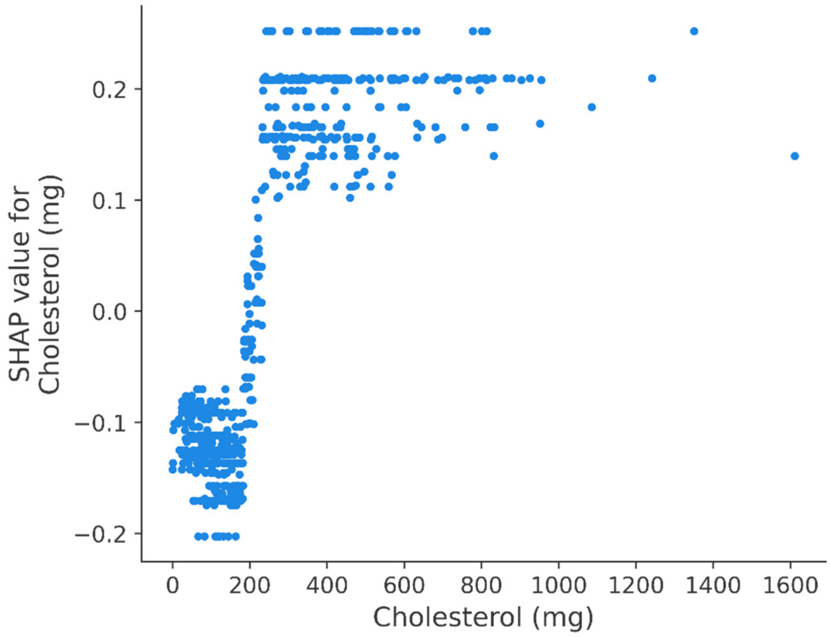
SHAP value for
Cholesterol (mg)
0.2
0.1
0.0
−0.1
−0.2
0
200
400
600
800
1000
1200
1400
1600
Cholesterol (mg)

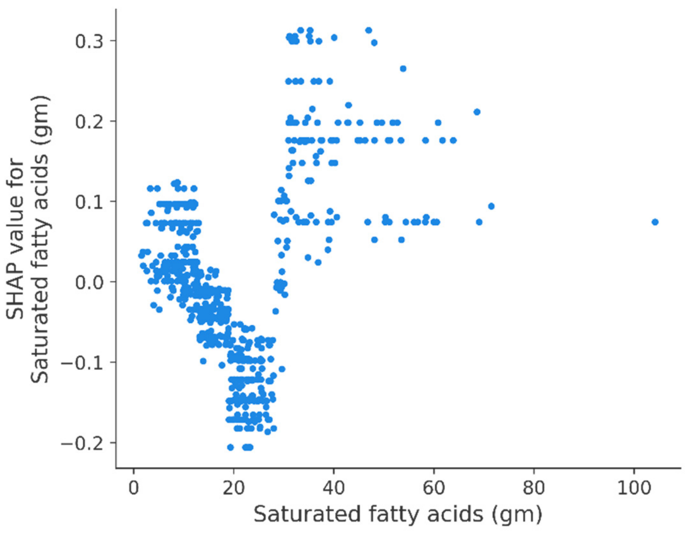
SHAP value for
Saturated fatty acids (gm)
0.3
0.2
0.1
0.0
−0.1
−0.2
0
20
40
60
80
100
Saturated fatty acids (gm)

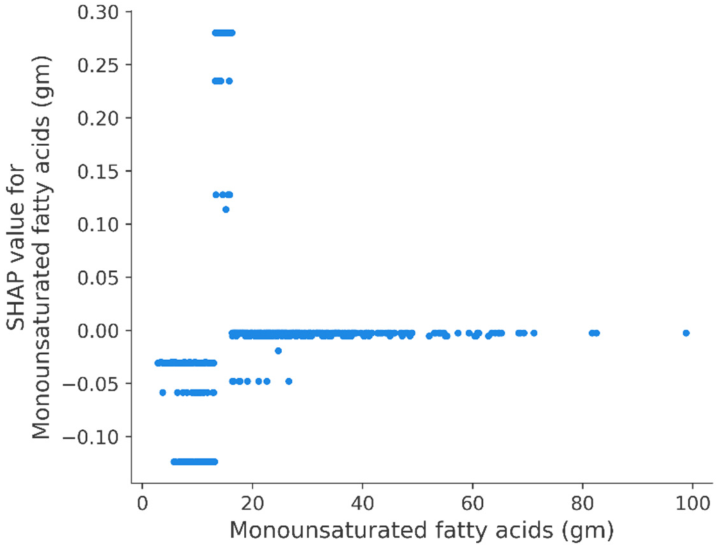
SHAP value for
Monounsaturated fatty acids (gm)
0.30
0.25
0.20
0.15
0.10
0.05
0.00
−0.05
−0.10
0
20
40
60
80
100
Monounsaturated fatty acids (gm)

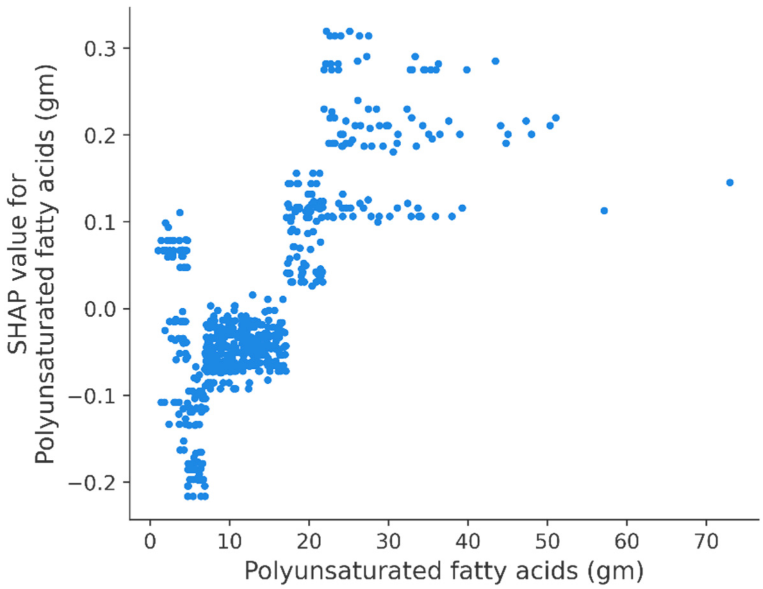
SHAP value for
Polyunsaturated fatty acids (gm)
0.3
0.2
0.1
0.0
−0.1
−0.2
0
10
20
30
40
50
60
70
Polyunsaturated fatty acids (gm)

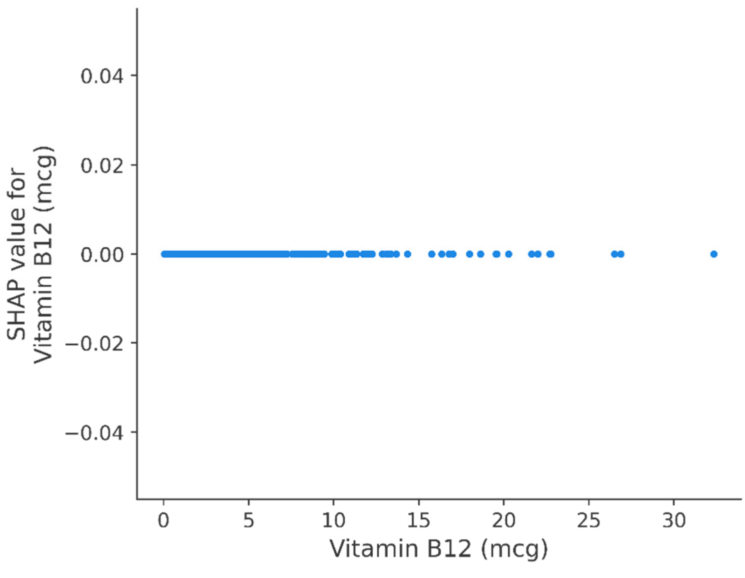
SHAP value for
Vitamin B12 (mcg)
0.04
0.02
0.00
−0.02
−0.04
0
5
10
15
20
25
30
Vitamin B12 (mcg)

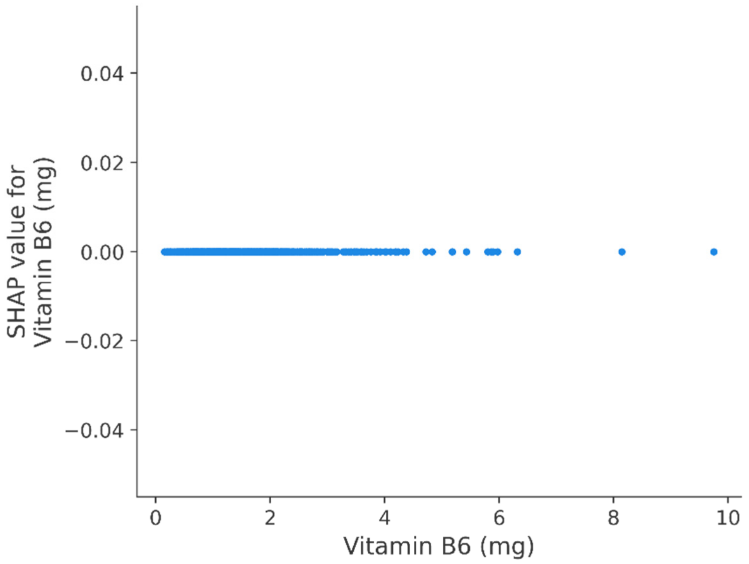
0.04
0.02
0.00
−0.02
−0.04
SHAP value for Vitamin B6 (mg)
0
2
4
6
8
10
Vitamin B6 (mg)

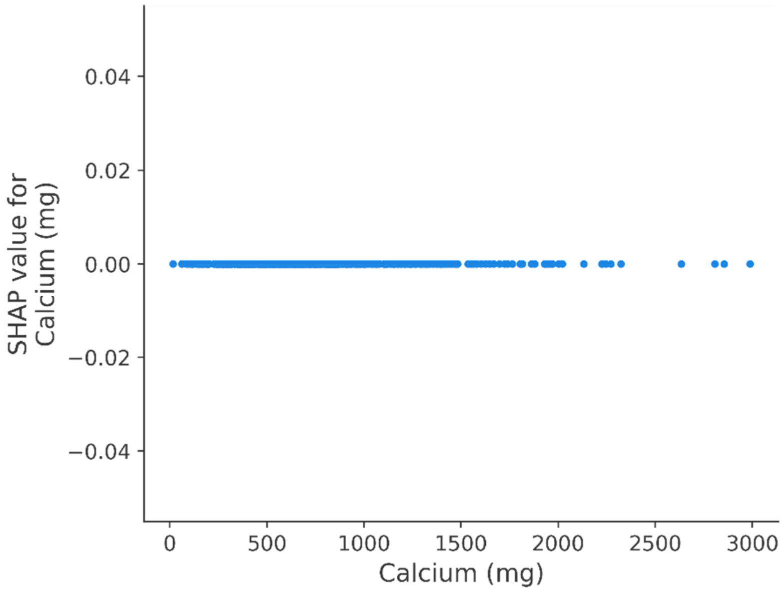
0.04
0.02
0.00
−0.02
−0.04
SHAP value for Calcium (mg)
0
500
1000
1500
2000
2500
3000
Calcium (mg)

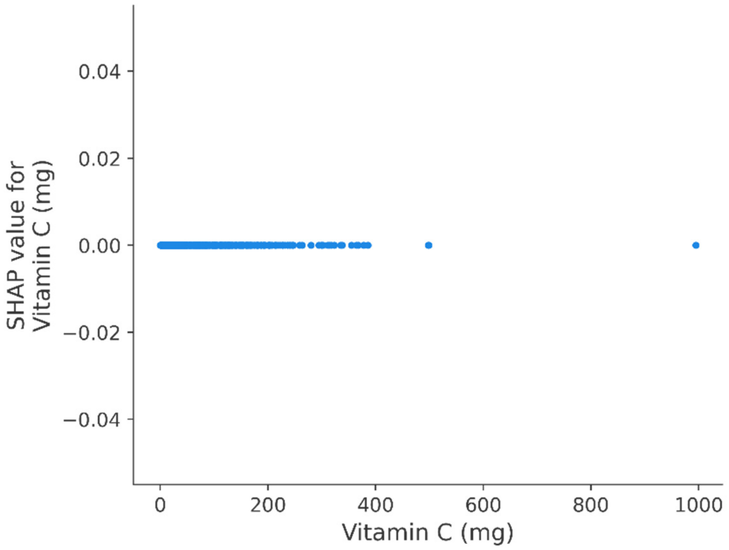
0.04
0.02
0.00
−0.02
−0.04
SHAP value for Vitamin C (mg)
0
200
400
600
800
1000
Vitamin C (mg)

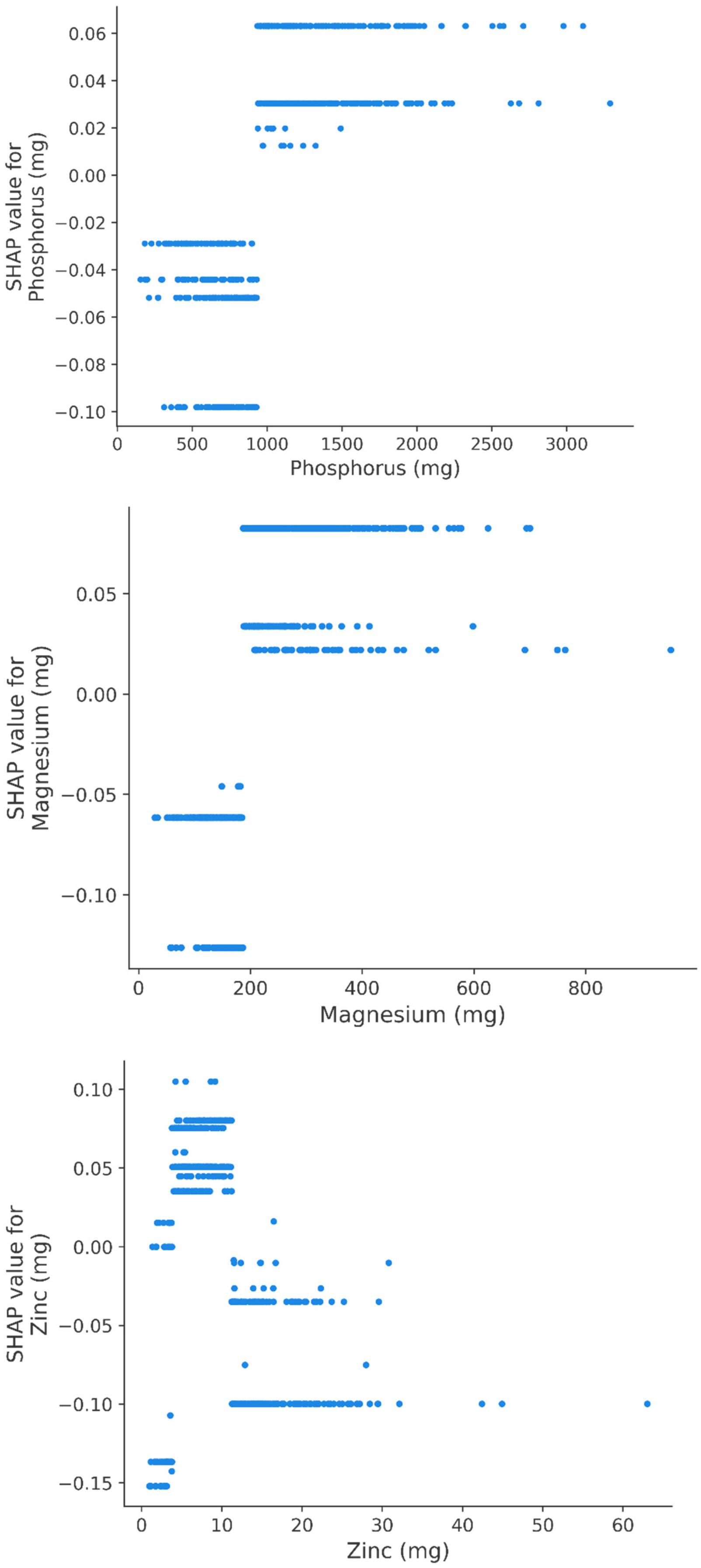
SHAP value for Phosphorus (mg)
Phosphorus (mg)
0.06
0.04
0.02
0.00
−0.02
−0.04
−0.06
−0.08
−0.10
0
500
1000
1500
2000
2500
3000
SHAP value for Magnesium (mg)
Magnesium (mg)
0.05
0.00
−0.05
−0.10
0
200
400
600
800
SHAP value for Zinc (mg)
Zinc (mg)
0.10
0.05
0.00
−0.05
−0.10
−0.15
0
10
20
30
40
50
60

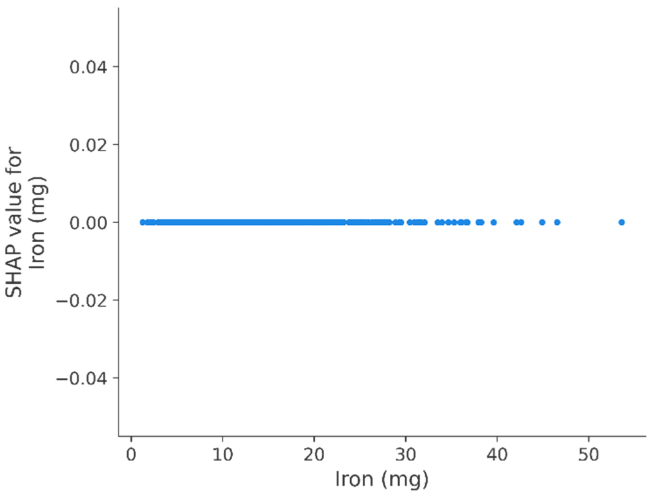

SHAP value for
Iron (mg)
0.04
0.02
0.00
−0.02
−0.04
0
10
20
30
40
50
Iron (mg)

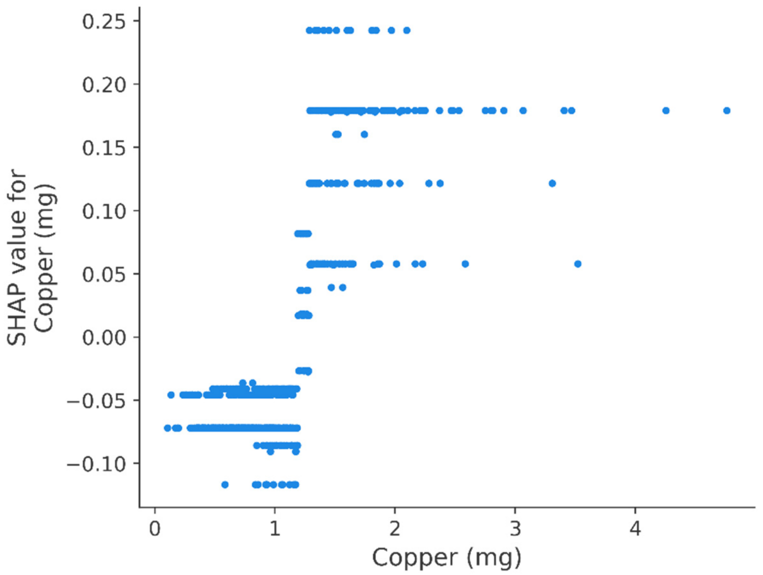

SHAP value for
Copper (mg)
0.25
0.20
0.15
0.10
0.05
0.00
−0.05
−0.10
0
1
2
3
4
Copper (mg)

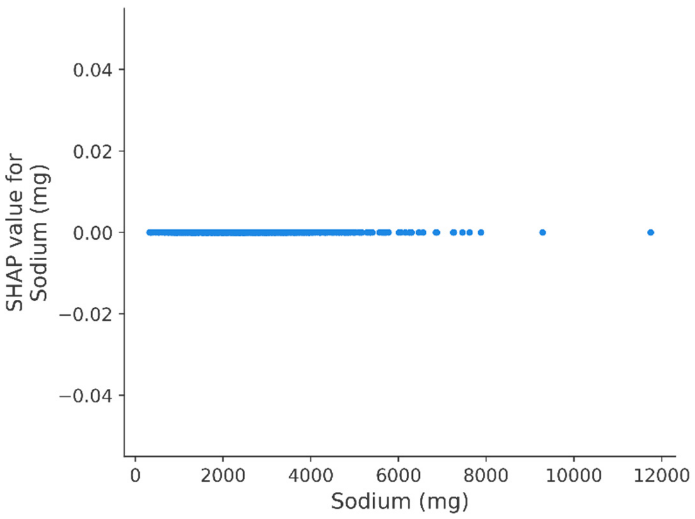

SHAP value for
Sodium (mg)
0.04
0.02
0.00
−0.02
−0.04
0
2000
4000
6000
8000
10000
12000
Sodium (mg)

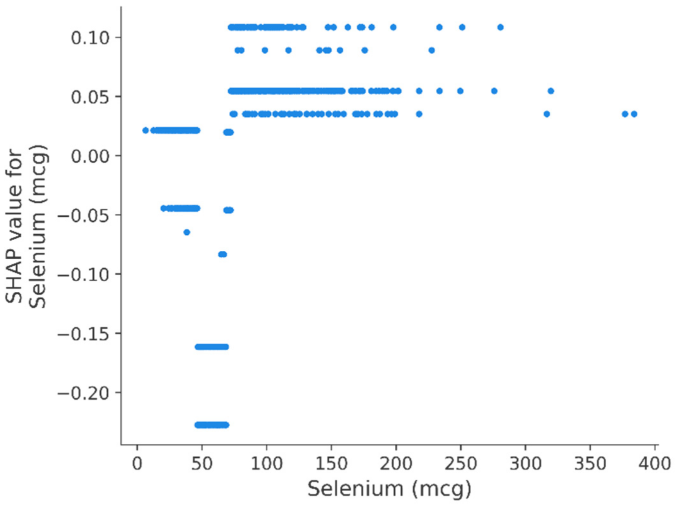
SHAP value for
Selenium (mcg)
0.10
0.05
0.00
−0.05
−0.10
−0.15
−0.20
0
50
100
150
200
250
300
350
400
Selenium (mcg)

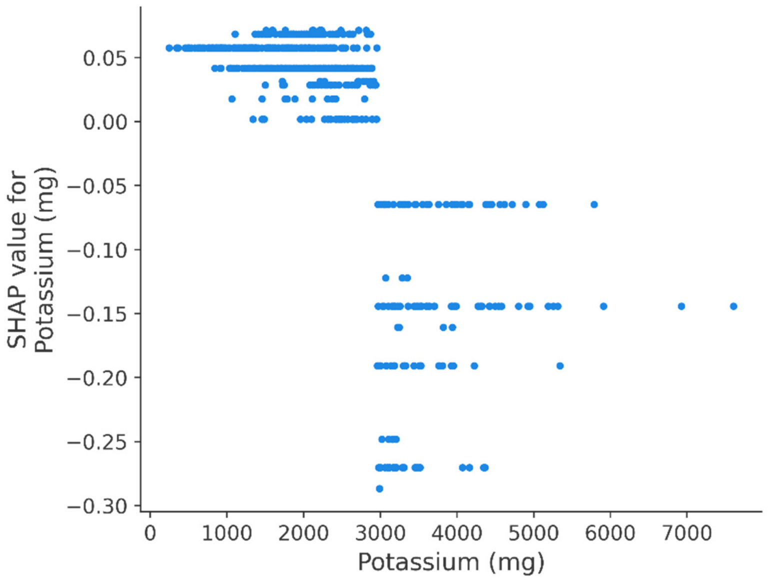
SHAP value for
Potassium (mg)
0.05
0.00
−0.05
−0.10
−0.15
−0.20
−0.25
−0.30
0
1000
2000
3000
4000
5000
6000
7000
Potassium (mg)

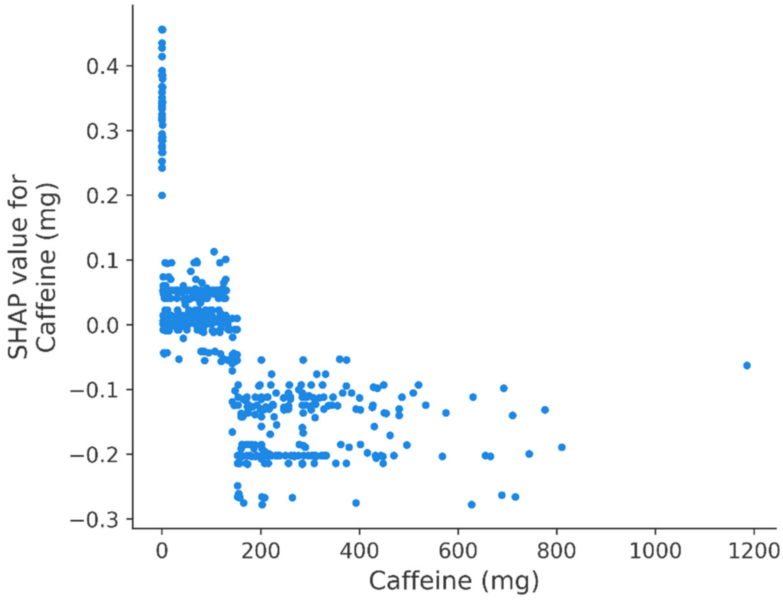
SHAP value for
Caffeine (mg)
0.4
0.3
0.2
0.1
0.0
−0.1
−0.2
−0.3
0
200
400
600
800
1000
1200
Caffeine (mg)

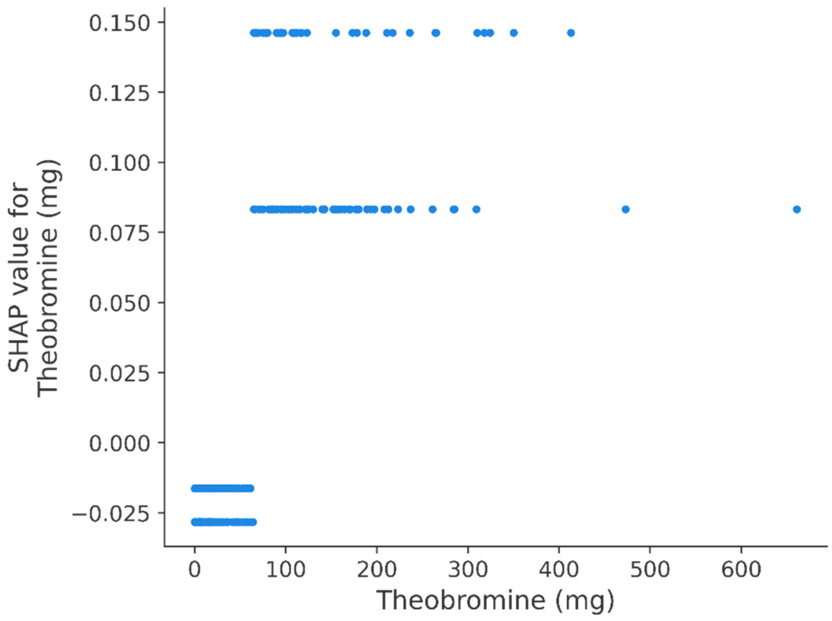
0.150
0.125
0.100
0.075
0.050
0.025
0.000
−0.025
SHAP value for
Theobromine (mg)
0
100
200
300
400
500
600
Theobromine (mg)

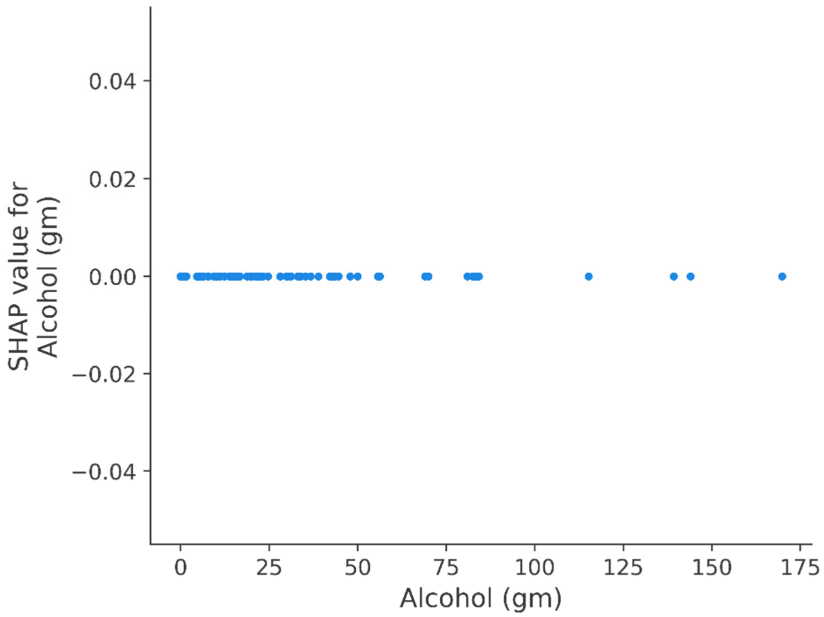
0.04
0.02
0.00
−0.02
−0.04
SHAP value for
Alcohol (gm)
0
25
50
75
100
125
150
175
Alcohol (gm)

Figure S2. SHAP values of all features from RF algorithm

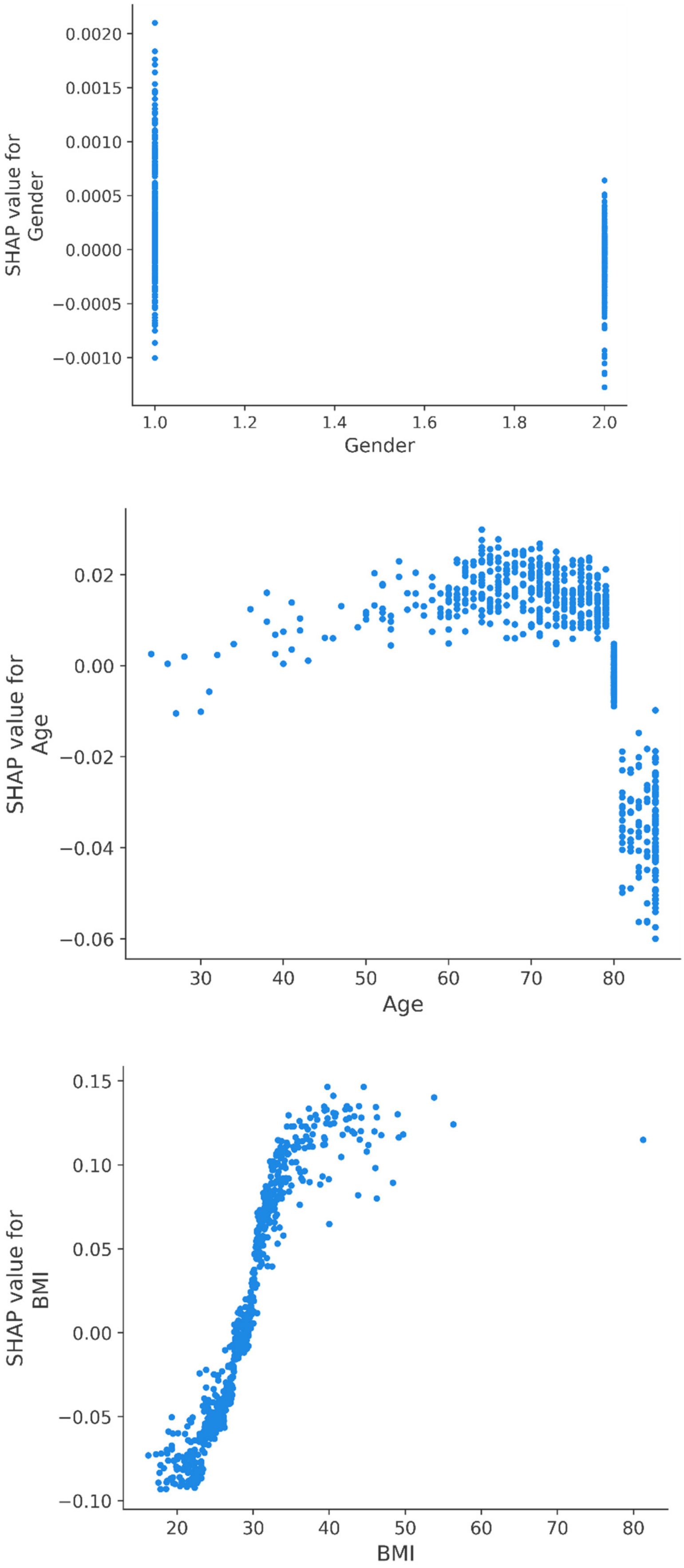

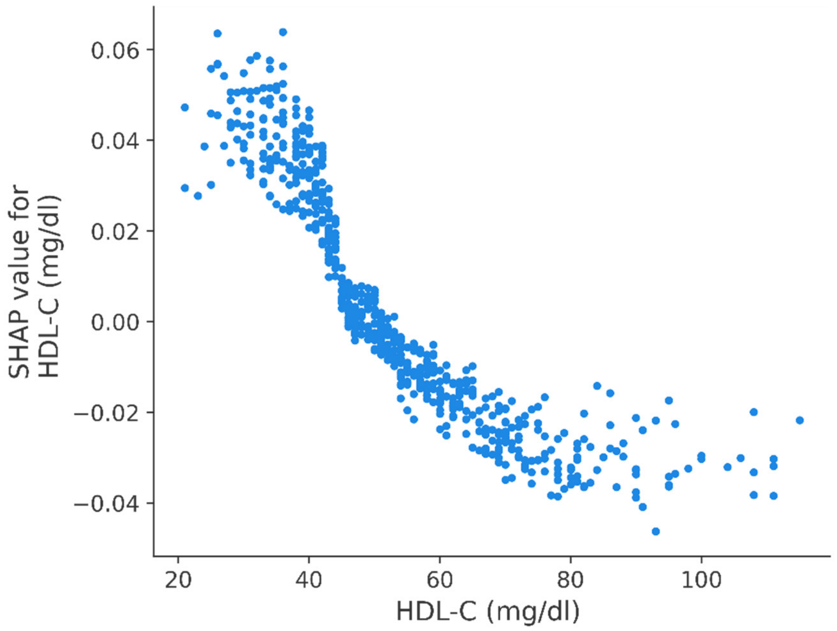
SHAP value for
HDL-C (mg/dl)
0.06
0.04
0.02
0.00
−0.02
−0.04
20
40
60
80
100
HDL-C (mg/dl)

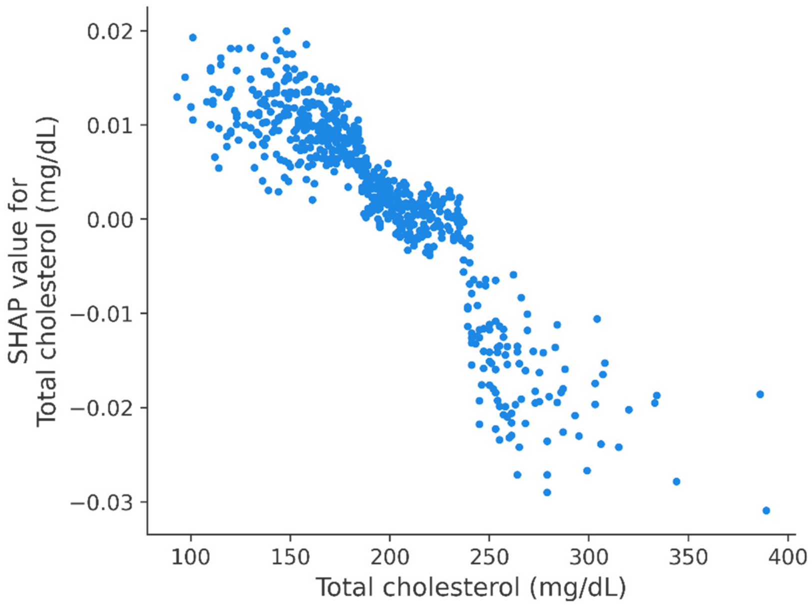
SHAP value for
Total cholesterol (mg/dL)
0.02
0.01
0.00
−0.01
−0.02
−0.03
100
150
200
250
300
350
400
Total cholesterol (mg/dL)

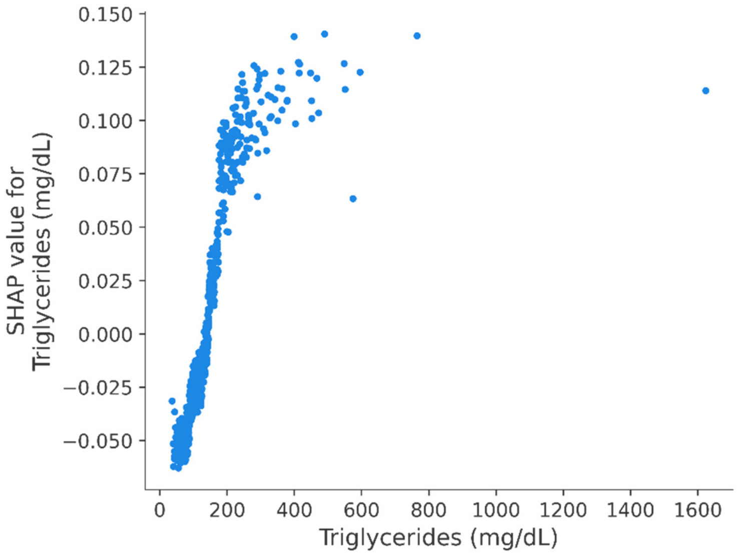
SHAP value for
Triglycerides (mg/dL)
0.150
0.125
0.100
0.075
0.050
0.025
0.000
−0.025
−0.050
0
200
400
600
800
1000
1200
1400
1600
Triglycerides (mg/dL)

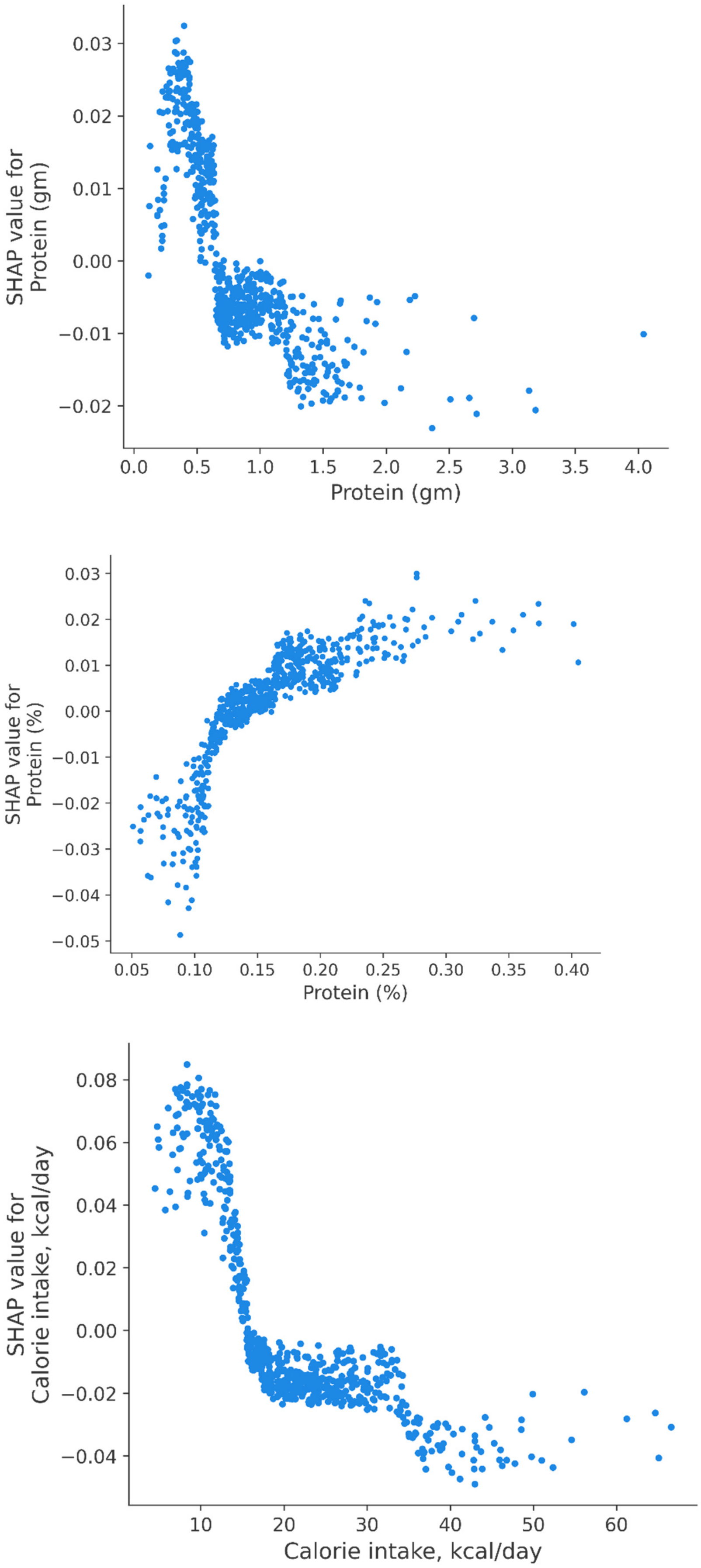
SHAP value for
Protein (gm)
0.03
0.02
0.01
0.00
−0.01
−0.02
0.0
0.5
1.0
1.5
2.0
2.5
3.0
3.5
4.0
Protein (gm)
SHAP value for
Protein (%)
0.03
0.02
0.01
0.00
−0.01
−0.02
−0.03
−0.04
−0.05
0.05
0.10
0.15
0.20
0.25
0.30
0.35
0.40
Protein (%)
SHAP value for
Calorie intake, kcal/day
0.08
0.06
0.04
0.02
0.00
−0.02
−0.04
10
20
30
40
50
60
Calorie intake, kcal/day

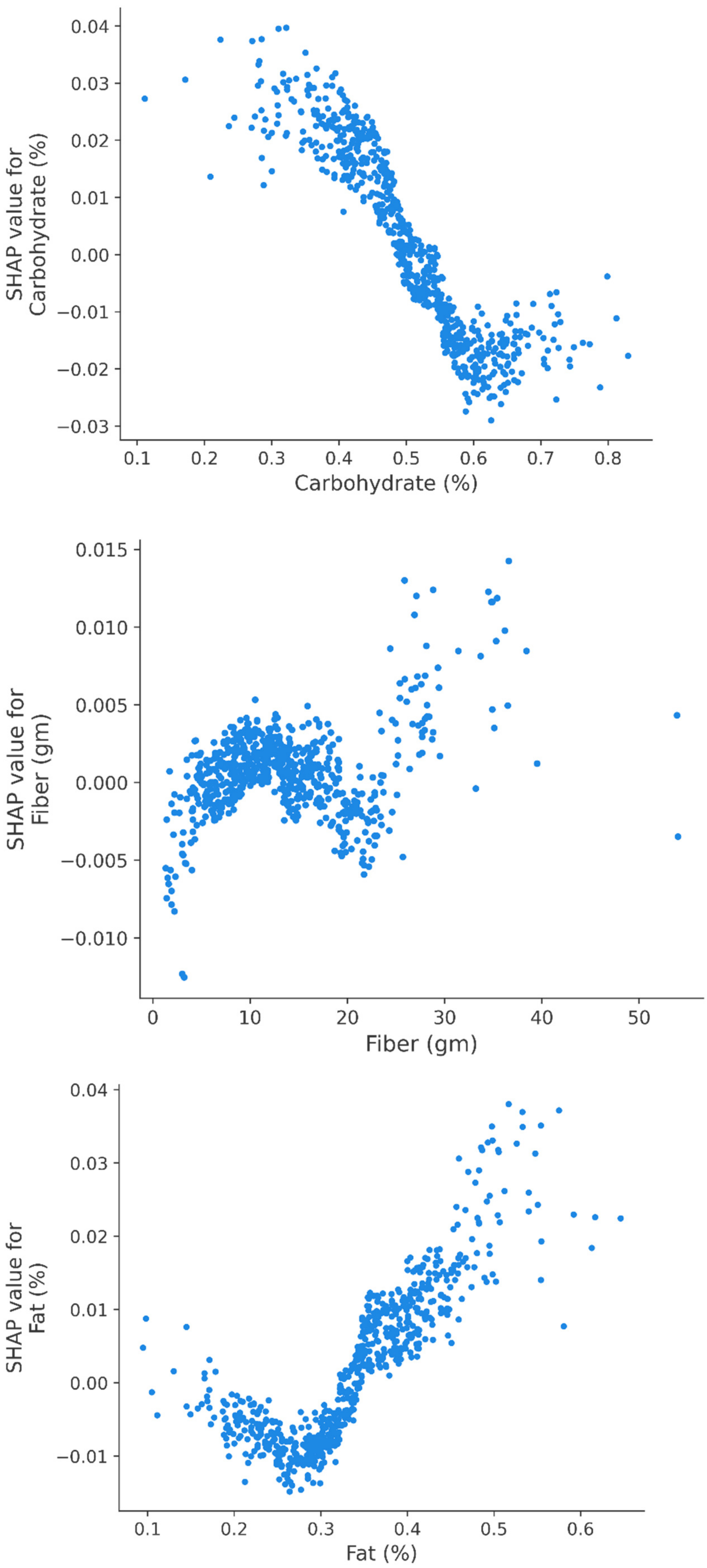

0.04
0.03
0.02
0.01
0.00
−0.01
−0.02
−0.03
SHAP value for Carbohydrate (%)
0.1 0.2 0.3 0.4 0.5 0.6 0.7 0.8
Carbohydrate (%)
0.015
0.010
0.005
0.000
−0.005
−0.010
SHAP value for Fiber (gm)
0 10 20 30 40 50
Fiber (gm)
0.04
0.03
0.02
0.01
0.00
−0.01
SHAP value for Fat (%)
0.1 0.2 0.3 0.4 0.5 0.6
Fat (%)

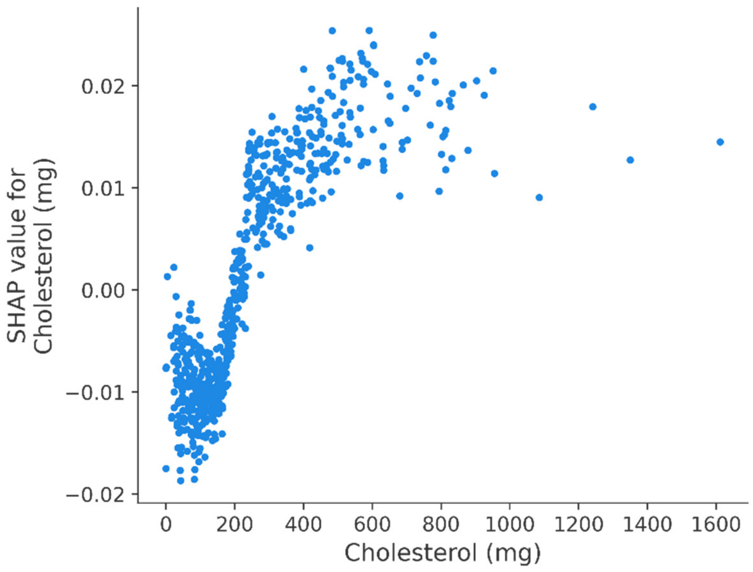
SHAP value for Cholesterol (mg)
0.02
0.01
0.00
−0.01
−0.02
0
200
400
600
800
1000
1200
1400
1600
Cholesterol (mg)

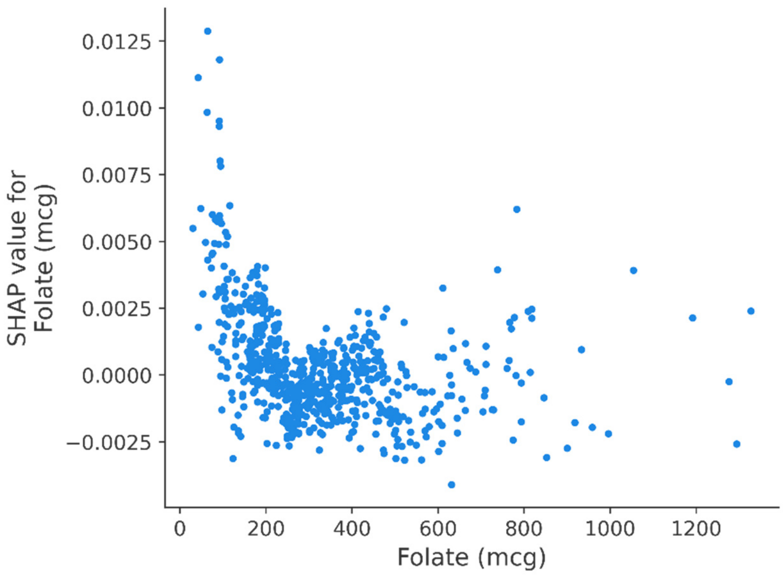
SHAP value for Folate (mcg)
0.0125
0.0100
0.0075
0.0050
0.0025
0.0000
−0.0025
0
200
400
600
800
1000
1200
Folate (mcg)

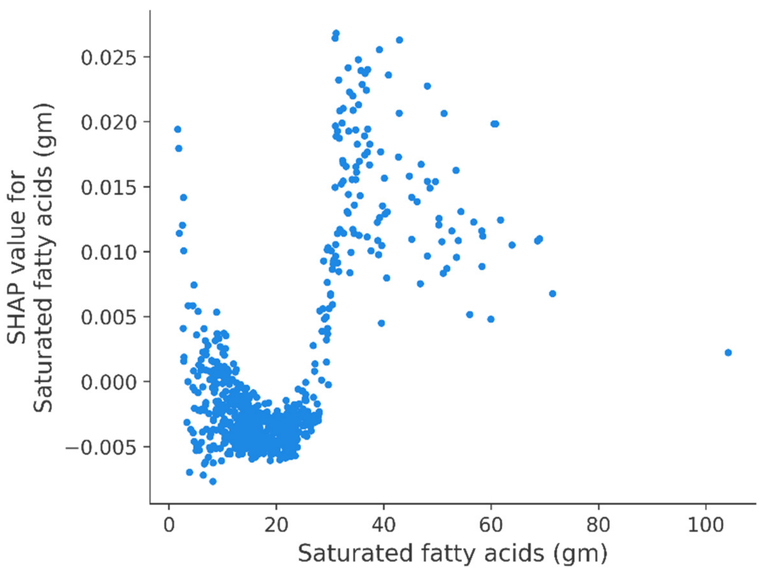
SHAP value for Saturated fatty acids (gm)
0.025
0.020
0.015
0.010
0.005
0.000
−0.005
0
20
40
60
80
100
Saturated fatty acids (gm)

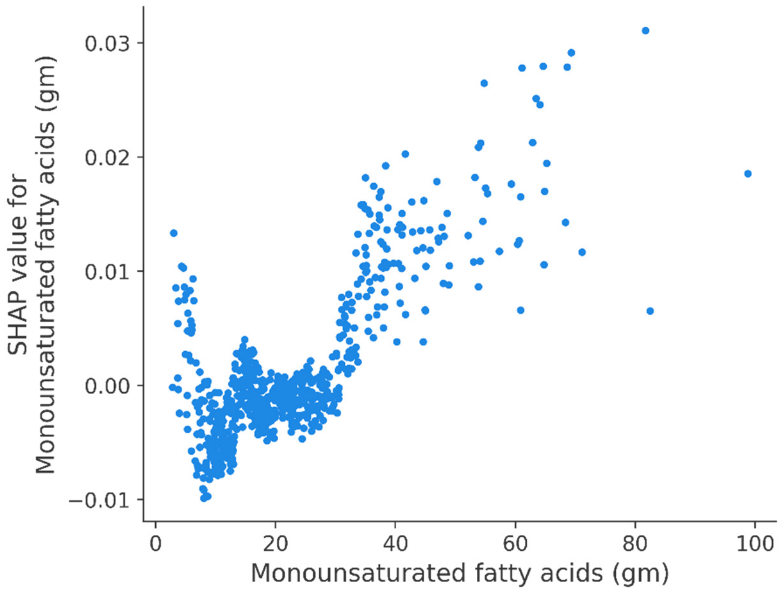
SHAP value for
Monounsaturated fatty acids (gm)
0.03
0.02
0.01
0.00
−0.01
0
20
40
60
80
100
Monounsaturated fatty acids (gm)

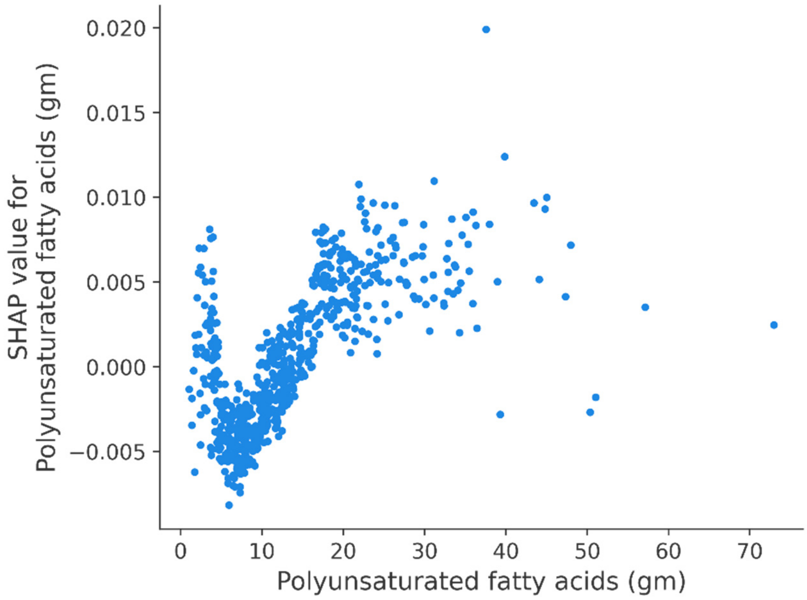
SHAP value for
Polyunsaturated fatty acids (gm)
0.020
0.015
0.010
0.005
0.000
−0.005
0
10
20
30
40
50
60
70
Polyunsaturated fatty acids (gm)

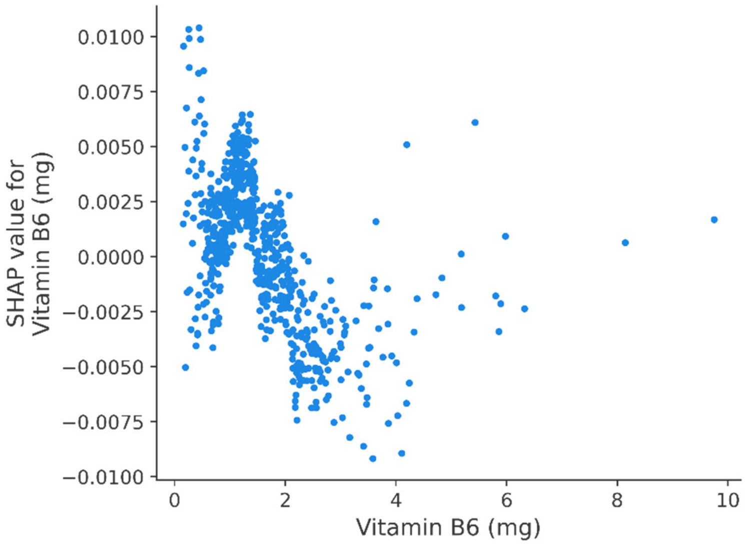
SHAP value for
Vitamin B6 (mg)
0.0100
0.0075
0.0050
0.0025
0.0000
−0.0025
−0.0050
−0.0075
−0.0100
0
2
4
6
8
10
Vitamin B6 (mg)

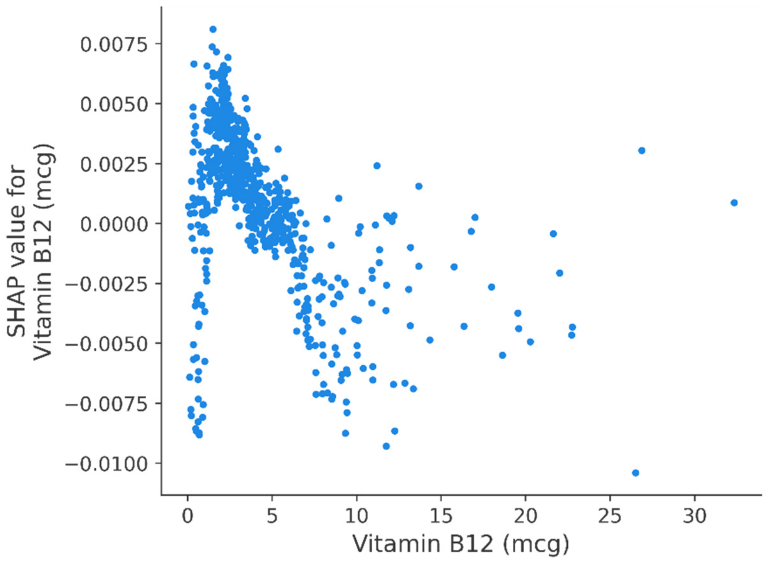

SHAP value for
Vitamin B12 (mcg)
0.0075
0.0050
0.0025
0.0000
−0.0025
−0.0050
−0.0075
−0.0100
0
5
10
15
20
25
30
Vitamin B12 (mcg)

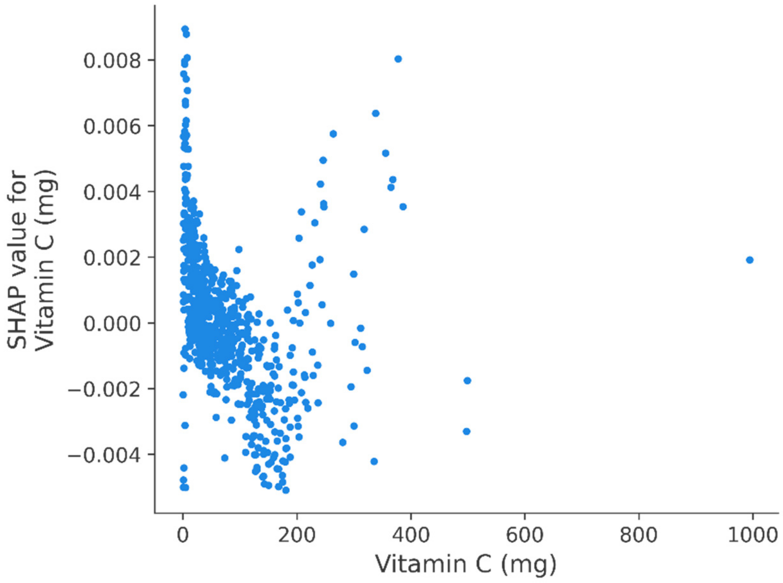

SHAP value for
Vitamin C (mg)
0.008
0.006
0.004
0.002
0.000
−0.002
−0.004
0
200
400
600
800
1000
Vitamin C (mg)

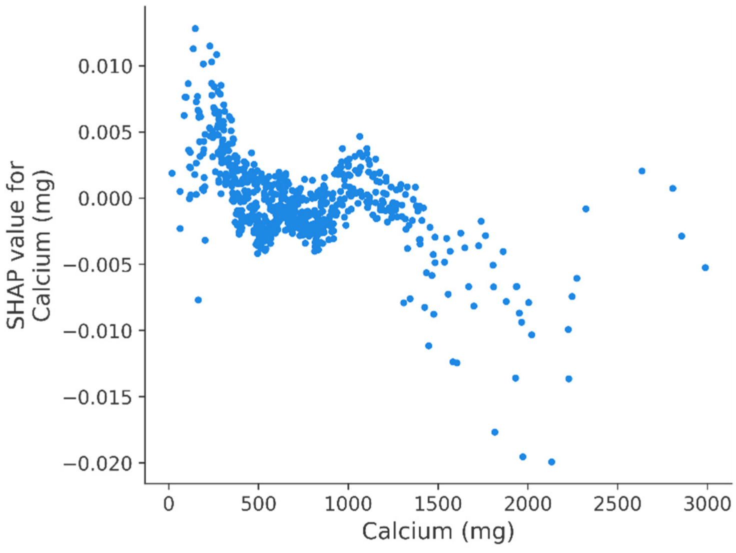

SHAP value for
Calcium (mg)
0.010
0.005
0.000
−0.005
−0.010
−0.015
−0.020
0
500
1000
1500
2000
2500
3000
Calcium (mg)

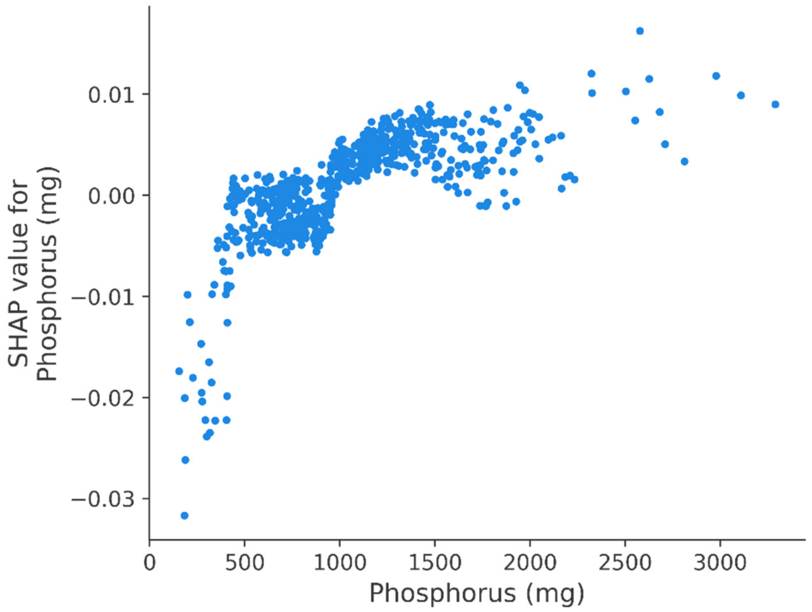

SHAP value for Phosphorus (mg)
0.01
0.00
−0.01
−0.02
−0.03
0
500
1000
1500
2000
2500
3000
Phosphorus (mg)

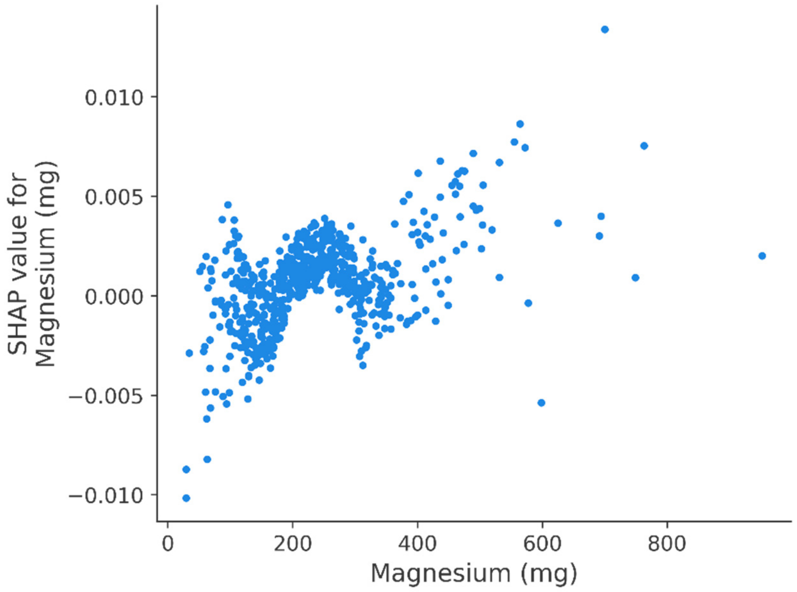

SHAP value for Magnesium (mg)
0.010
0.005
0.000
−0.005
−0.010
0
200
400
600
800
Magnesium (mg)

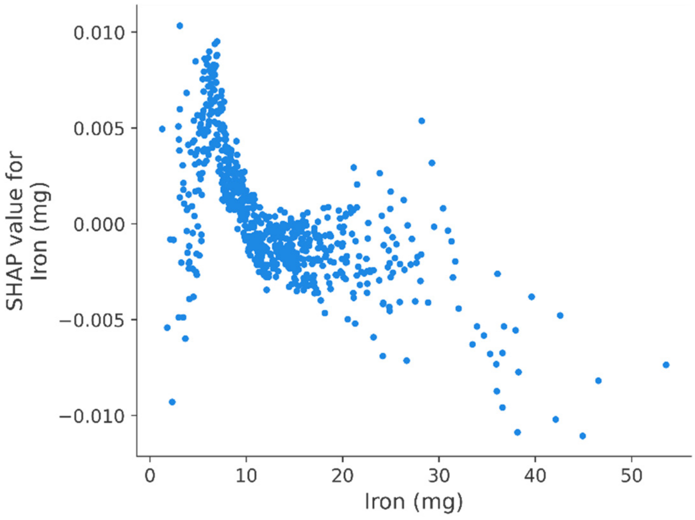

SHAP value for Iron (mg)
0.010
0.005
0.000
−0.005
−0.010
0
10
20
30
40
50
Iron (mg)

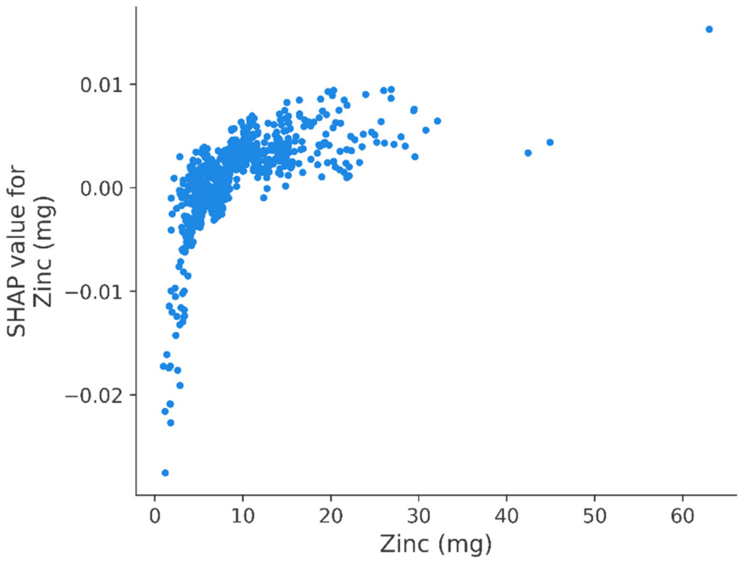

SHAP value for Zinc (mg)
0.01
0.00
−0.01
−0.02
0
10
20
30
40
50
60
Zinc (mg)

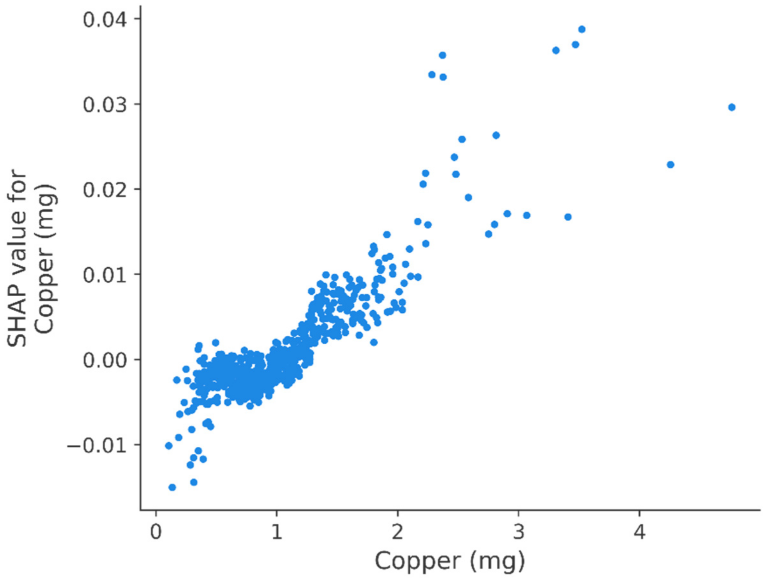

SHAP value for Copper (mg)
0.04
0.03
0.02
0.01
0.00
−0.01
0
1
2
3
4
Copper (mg)

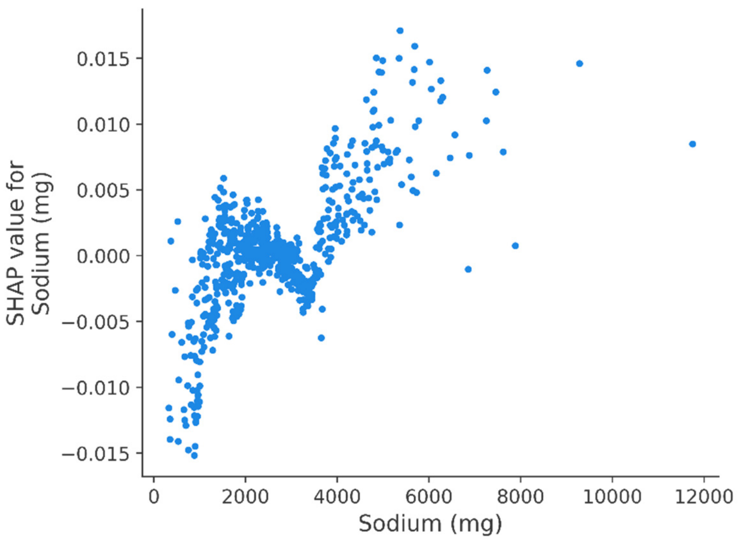

SHAP value for Sodium (mg)
0.015
0.010
0.005
0.000
−0.005
−0.010
−0.015
0
2000
4000
6000
8000
10000
12000
Sodium (mg)

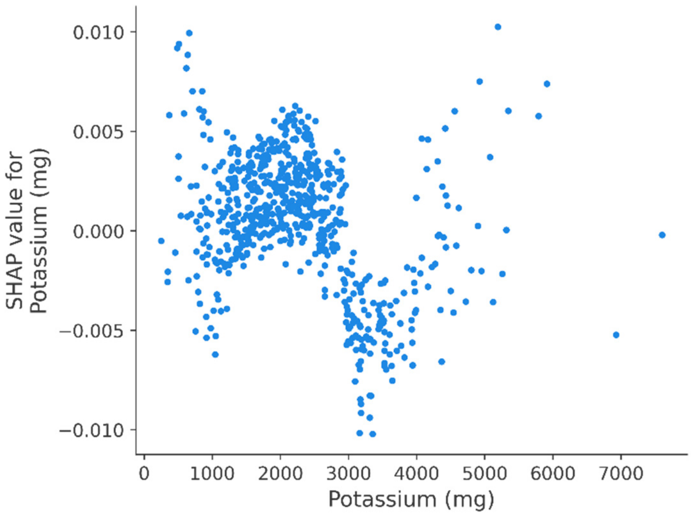
SHAP value for Potassium (mg)
Potassium (mg)
0.010
0.005
0.000
−0.005
−0.010
0
1000
2000
3000
4000
5000
6000
7000

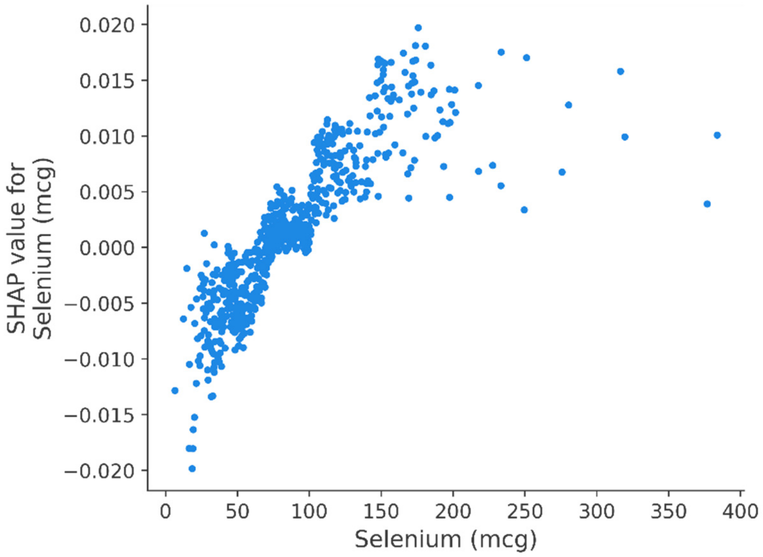
SHAP value for Selenium (mcg)
Selenium (mcg)
0.020
0.015
0.010
0.005
0.000
−0.005
−0.010
−0.015
−0.020
0
50
100
150
200
250
300
350
400

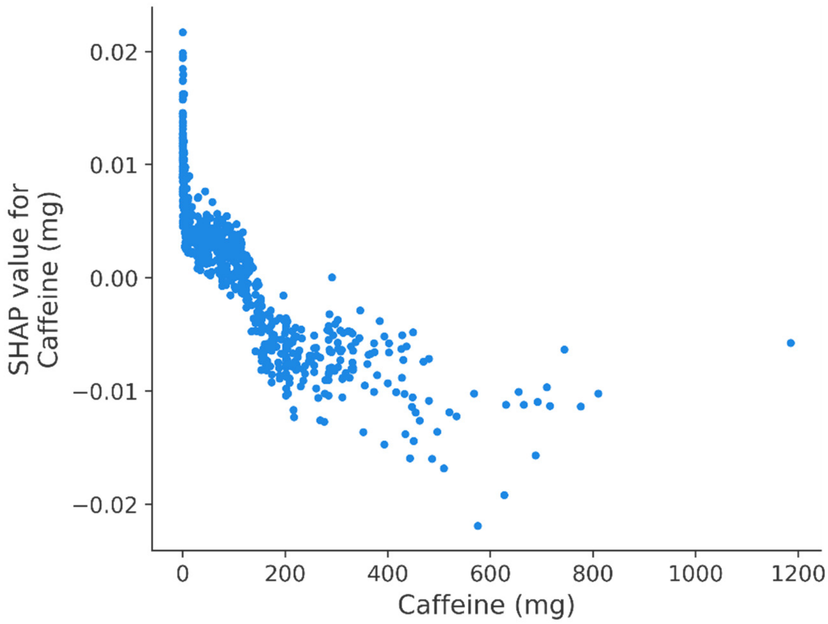
SHAP value for Caffeine (mg)
Caffeine (mg)
0.02
0.01
0.00
−0.01
−0.02
0
200
400
600
800
1000
1200

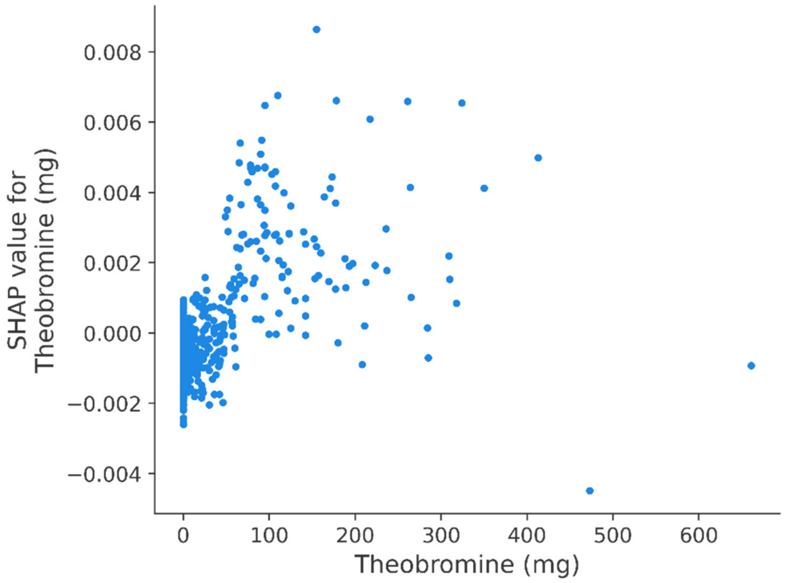
0.008
0.006
0.004
0.002
0.000
−0.002
−0.004
SHAP value for
Theobromine (mg)
0
100
200
300
400
500
600
Theobromine (mg)

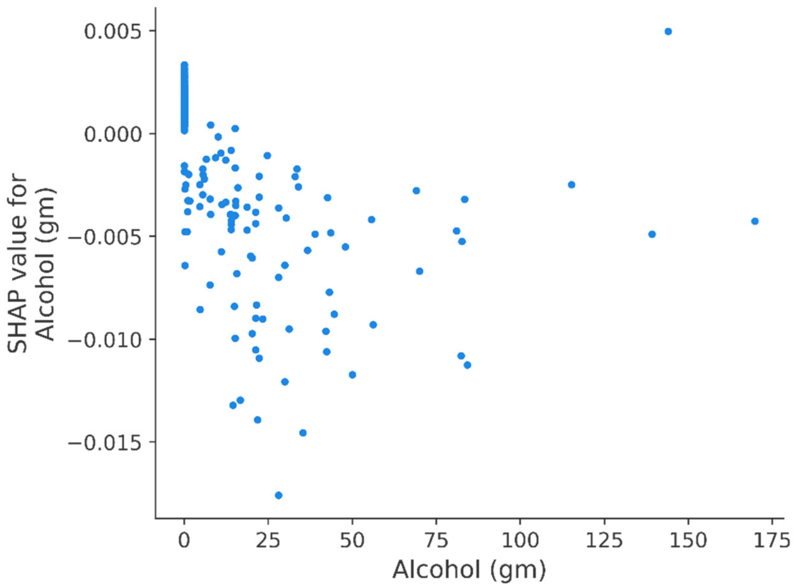
0.005
0.000
−0.005
−0.010
−0.015
SHAP value for
Alcohol (gm)
0
25
50
75
100
125
150
175
Alcohol (gm)
